# Adherence to ketoacids/essential amino acids-supplemented low protein diets and new indications for patients with chronic kidney disease

**DOI:** 10.1186/s12882-016-0278-7

**Published:** 2016-07-07

**Authors:** Denis Fouque, Jing Chen, Wei Chen, Liliana Garneata, SJ Hwang, Kamyar Kalantar-Zadeh, Joel D. Kopple, William E. Mitch, Giorgina Piccoli, Vladimir Teplan, Philippe Chauveau

**Affiliations:** UCBL, Centre Hospitalier Lyon-Sud, University Lyon, Carmen, Cens, F-69622 Lyon, France; Division of Nephrology, Huashan Hospital, Fudan University, Shanghai, China; Department of Parenteral and Enteral Nutrition, Peking Union Medical College Hospital, Beijing, China; Department Nephrology and Internal Medicine, Dr Carol Davila Hospital of Nephrology, Carol Davila University of Medicine and Pharmacy, Bucharest, Romania; Division Nephrology, Kaohsiung Medical University Hospital, Graduate Institute of Clinical Medicine, College of Medicine, Kaohsiung Medical University, Kaohsiung, Taiwan; Division Nephrology and Hypertension, University of California Irvine School of Medicine, Irvine, CA USA; Deparment Epidemiology, UCLA Fielding School of Public Health, Los Angeles, CA USA; Division Nephrology and Hypertension, Los Angeles Biomedical Research Institute at Harbor-UCLA Medical Center, Torrance, CA USA; David Geffen School of Medicine at UCLA, UCLA Fielding School of Public Health, Los Angeles, CA USA; Nephrology Division, Baylor College of Medicine, Houston, TX USA; SS Nephrology, ASOU San Luigi, Departmentt of Clinical and Biological Sciences, University of Torino, Turin, Italy; Department Nephrol, Institute Clin Exp Med, Transpl Centre, Prague, Czech Republic; Service de Néphrologie, CHU de Bordeaux & Aurad-Aquitaine, Bordeaux, France; Department Nephrology, Centre Hospitalier Lyon Sud, Chemin du grand revoyet, 69495 Pierre Bénite, France

**Keywords:** Protein, Ketoacid, Keto-analog, Low protein diet, Kidney disease, Wasting, Dialysis

## Abstract

**Background:**

Low protein diets (LPD) have long been prescribed to chronic kidney disease patients with the goals of improving metabolic abnormalities and postpone the start of maintenance dialysis.

**Methods:**

We reviewed the recent literature addressing low protein diets supplemented with ketoacids/essential aminoacids prescribed during chronic kidney disease and their effects on metabolic, nutritional and renal parameters since 2013.

**Results:**

We show new information on how to improve adherence to these diets, on metabolic improvement and delay of the dialysis needs, and preliminary data in chronic kidney disease associated pregnancy. In addition, data on incremental dialysis have been reviewed, as well as potential strategies to reverse protein energy wasting in patients undergoing maintenance dialysis.

**Conclusion:**

These recent data help to better identify the use of low protein diets supplemented with ketoacids/essential aminoacids during chronic kidney disease.

## Background

Maintenance dialysis is a burden for the global health systems globally and is growing at an unprecedented rate. A recent analysis of the prevalence of end stage renal disease led to the conclusion that the prevalence of maintenance dialysis has grown more rapidly during the last two decades than predicted [1]. Efforts to delay the progression to end stage renal disease (ESRD) mainly rely on control of blood pressure and diabetes. Unfortunately, in many countries nephrologists generally do not examine CKD patients until dialysis is imminent and they have few options for delaying the time to dialysis. However, increasing the time until transition to dialysis therapy may improve patient’s quality of life and can reduce the financial strain on the healthcare system. Consequently, postponing dialysis initiation should receive a high priority in majority of patients with advanced CKD. In fact, there is evidence that many patients will be able to postpone the transition to dialysis, reportedly, more than 60 % of all CKD patients can experience a persistently low GFR (i.e., below 25 ml/min/1.73 m^2^) during the two years prior to initiation of dialysis [2, 3]. In short, there is opportunity to reduce the drivers of chronic kidney disease, including poorly controlled hypertension and decreased symptoms of uremia using a low protein diet supplemented with ketoanalogues in countries where these agents are available and including dietary salt restriction [4, 5]. In jurisdictions without approved ketoacids such as the United States or Canada, essential amino acids can be used instead. These strategies were concisely reviewed in 2013 [6], therefore this review will focus on evidence for efficacy that has become available since. In addition, it should be recalled that the currently available ketoanalogues (KA) preparations are marketed with other essential amino acids (EAA), which will be referred as KA/EAA below in text.

## How to improve adherence and select patients to a KA/EAA-supplemented low protein diet

The general approach to treatment of patients with low or supplemented very low protein diets often results in a tug of war between the patient’s will to comply vs nutritional temptations and deeply rooted food habits. Consequently, doctors should consider that to attain good compliance it is advisable to set goals of adherence – the conscious decision of the patient to stick with a therapy – as a therapeutic goal. To overcome this often frustrating situation, Piccoli et al. [7] sought to identify factors that influence patient adherence. They investigated implementation of a simplified low-protein, KA/EAA supplemented diet as part of the routine work-up of CKD patients. Patients with CKD stages 4 to 5 or patients with CKD stage 3 and rapid progression and/or a refractory nephrotic syndrome were offered SLPD (0.6 g protein/kg/day). The endpoint was defined as at least six months of follow-up on the diet and the results were analysed by comparing demographic and health parameters among patients following the diet for less than one month vs. those who followed the restricted diet for up to six months. The results indicated that successful adherence was unpredictable a priori, as no medical or social parameters were identified as indicative of continuous adherence to the SLPD [7]. Renal nutrition education has also been reported to improve adherence to LPD in a Brazilian randomized controlled trial [8].

Another recent study [9] from the same group addressed the compliance issue in 185 patients given KA/EAA supplemented LPD (0.6 g protein/kg/day supplemented with 1 tablet per 10 kg BW/day) vs 122 patients prescribed a LPD with the same protein intake and commercially available protein-free food. Adherence was assessed from food questionnaires, 24 h urine urea collection and tablet counts. Patients who chose SLPD were younger and had significantly lower GFR values and higher levels of proteinuria. After six months, the median protein intake was 0.7 g/kg BW/d in both groups. Albumin and total protein levels were stable at six and twelve months. Of note, even after GFR fell below 15 ml/min, the diet was still followed by 59 % patients at six months and 32 % at twelve months. At one year, all patients had reduced their albuminuria and degree of acidemia. Interestingly the two low protein diets were comparable in cost. Thus, compared to an “early start” dialysis (i.e., when GFR is >10 ml/min), this dietary approach of an KA/EAA supplemented 0.6 g protein/kg/day diet resulted in substantial financial savings [10].

A landmark trial for the therapy with supplemented low protein diet by Garneata et al. has been recently published [11]. These investigators conducted a prospective, open, parallel, randomized controlled study comparing vegetarian VLPD (0.3 g/kg/day) supplemented with KA/EAA, 1 tablet/5 kg BW with LPD (0.6 g/kg/day) in non-diabetic patients with a stable eGFR below 30 ml/min/1.73 m^2^. The study had a three-step enrolment procedure: patients were asked if they would be willing to follow a low protein diet, vegetarian if necessary; only those who were willing to do so were included into a three month run-in period with a low protein diet. 1,413 patients were assessed for eligibility and 42 % of them declined to consider a vegetarian diet. Dietary compliance was controlled monthly, and only patients who followed the diet were finally enrolled and followed for up to 18 months. In the run-in phase, another 44 % dropped out, mainly for non-adherence to the LPD. Thus only 14 % (207) of the patients initially eligible entered the intervention period. The primary composite endpoint was initiation of dialysis or a reduction in the initial eGFR by more than 50 %. The SVLPD resulted in a significant delay of time to dialysis: while only 13 % in the SVLPD group reached the primary endpoint, 42 % did in the non-supplemented LPD group. Mean time to event was longer in the SVLPD group with 57 (55–59) weeks vs 47 (43–50) weeks in the control group (*p* < 0.001). The decline in eGFR was slower in SVLPD group as compared to LPD with a median difference between groups of 3.2 [2.6–3.8] mL/min. At 15 months, only the SVLPD group experienced a significant reduction of serum urea (120 [84–132] vs 167 [136–273] mg/dL) and serum phosphorus (4.4 [4.1–4.8] vs 5.5 [4.3–6.9,] mg/dl) while serum calcium (4.5 [4.4–4.7] vs 3.9 (3.9–4.0), mg/dL) and serum bicarbonate increased significantly (23.9 [21.5–25.1] vs 17.3 [15.3–18.7] mEq/L). The adjusted “number needed to treat” (NNT) to avoid the primary composite endpoint was 4.0 (3.9–4.4). The adjusted NNT to avoid dialysis initiation was 22.4 (21.5–25.1), but decreased to 2.7 (2.6–3.1) when only patients with eGFR <20 mL/min were retained in analysis. No significant changes in nutritional status and no drug-related adverse reactions were noted. Elderly (>65 years) and young active patients (<45 years) seemed to more easily accept the dietary intervention. Strong family/social support and, being already vegan or vegetarian were also identified as supportive characteristics [11]. Thus, adherence to a SVLPD can be achieved only if a good selection strategy is made and test periods are performed. Overall however, only 30 % of the pre-selected patients correctly achieved the very low protein intake reduction.

## New features of low protein diets in chronic kidney disease and dialysis

### Pregnancy

It has long been feared that provision of a LPD to pregnant patients with CKD could interfere with growth of the foetus. Piccoli et al. [12]. assessed the effect of a vegan-based LPD supplemented with KA/EAA on fetal growth in pregnant women with CKD. In an open, interventional study of pregnant patients with stage 3–5 CKD or with proteinuria (>1 g/d in the first trimester or the presence of nephrotic-range proteinuria at any time), positive outcomes were observed during an evaluation from 2000 to 2012. Specifically, the patients treated with a LPD (0.6–0.8 g/kg per day) supplemented with KA/EAA plus 1 to 3 protein-unrestricted meals/week maintained a good nutritional status. In comparison to pregnant CKD women who did not receive LPD for various reasons (late referral, stable disease, previous nutritional problems), the women treated with protein-restricted-KA/EAA diets had fewer new-borns that were “small for gestational age” (i.e., 3 of 21 pregnancies) compared to those fed ad libitum (i.e., 7 of 16; *p* = 0.05) [12]. The mothers and children were followed for periods of six months to ten years and those treated with LPD-KA/EAA regimen had lower rates of hospitalization even though the prevalence of children below the third growth percentile were similar to events in the mothers fed ad libitum. These clinical outcomes certainly deserve to be tested in larger multicentre studies.

### Maturating arteriovenous fistulas

Can low protein diets help expanding time until initiation of dialysis? The best timing of dialysis initiation has long been a matter of debate and the IDEAL study [13] has proven that an early dialysis start may be hazardous. In addition, too often nephrologists do not become involved in the care of CKD patients until dialysis is imminent. Besides obvious advantages of an earlier referral of CKD patients to the nephrologist, late referrals may even interfere with the patients being introduced properly to dialysis (e.g., inadequate time to create a functional arteriovenous access or the option to be trained for peritoneal dialysis). Duenhas et al. [14] prospectively enrolled 21 patients who were scheduled to start haemodialysis based on serum urea reached ≥ 175 mg/dl and the creatinine clearance was ≤ 12 ml/min. These patients were advised to consume a “very low protein diet” (VLPD; 0.3 g/kg/day) consisting mainly of vegetable proteins and KA/EAA for 30 days to allow maturation of arteriovenous access and/or training for peritoneal dialysis. Ten of these patients (47.3 %) developed a matured permanent dialysis access within a mean follow up of 83.3 ± 58.2 days. Serum concentration of urea dropped significantly from a mean of 175.3 ± 48.3 mg/dl to 109.0 ± 25.8 mg/dl (*p* < 0.001). Serum calcium and phosphorus were improved while body mass index, muscle mass and other biochemical parameters including serum albumin were unchanged. The authors conclude that a very low protein diet supplemented with KA/EAA can maintain stable or even improved metabolic status in patients with very low renal function with no deterioration in protein-energy status until initiation of dialysis via a permanent dialysis access fistula [14]. These findings are of major clinical and public health importance because recent guidelines [15] call for an “intent-to-defer” as more efficacious compared to an “intent-to-start-early” approach in planning for the initiation of chronic dialysis in adults with an estimated GFR (eGFR) of less than 15 ml/min/1.73 m^2^. Authors of this consensus paper emphasize that better health-related quality of life and fewer burdens associated with earlier initiation of dialysis without clinical indications should be the goal, especially because some complications of uraemia may be avoided. In addition, this may help containing dialysis expenses as well [16].

### Incremental dialysis

Incremental dialysis should be discussed when searching for alternate dialysis initiation models. Indeed, the IDEAL trial [13] clearly showed that an early start of hemodialysis was of no benefit to patients, and as a consequence, an interest in incremental hemodialysis [17, 18] has recently arisen. This consists of one or two weekly hemodialysis sessions, based on the patient’s residual renal function and degree of metabolic abnormalities. As renal function decreases and/or metabolic disorders related to kidney failure increase, the patients dialysis dose increases until they begin a full thrice-weekly dialysis schedule. In a prospective non-randomized study of 85 Chinese MHD patients, after one year, those who were receiving twice dialysis sessions weekly did not show clinical or metabolic differences with patients under the regular thrice weekly regimen, except for residual diuresis which was better conserved in the twice-weekly group of patients [19]. In this setting, a prescription of a KA/EAA-supplemented very low protein diet may be considered to be an effective diet for maintaining a twice-weekly hemodialysis regimen for some more months and delay the loss of residual renal function [19], as it has also been reported in patients receiving peritoneal dialysis [20].

### Nutritional supplement during maintenance dialysis

It has been suggested that KA/EAA supplements might be considered for maintenance dialysis patients who present with protein energy wasting [21]. It has been showed that nutritional support, either enteral or intravenous, is able to reverse protein-energy wasting in wasted MHD patients [22]. This hypothesis, already suggested by Hiroshige et al. [23] who used branched-chain amino acids, has not yet been addressed by adequate clinical trials using KA/EAA and therefore should be a subject for future research. Preliminary data from a recent Chinese randomized trial in 100 chronic peritoneal dialysis patients, those patients who received KA/EAA supplements displayed reduced inflammatory status and a decreased serum leptin/adiponectin ratio as compared with the control group [24]. In addition, reducing protein intake with the addition of KA/EAA supplements, under strict dietary and nutritional assessments may help controlling serum phosphate in countries where limited phosphate binders are available [25, 26].

## Conclusion

Recent reports provide additional information that can be offered to advanced CKD patients, in order to help motivate them to adhere to these low and very low protein diets. These studies also provide additional evidence supporting the safety of these diets. Adherence to a protein-restricted diet can be improved by selecting patients and offering them tailored dietary choices. Taken together, these new research data confirm an overall picture that a low protein diet providing 0.3–0.6 g/kg BW/d with added KA/EAA supplements may improve proteinuria [27] and delays time until dialysis has to be started [28]. The latter most likely occurs because uremic toxicity is reduced.

## Abbreviations

CKD: Chronic kidney disease
ESRD: End-stage renal disease
EAA: Essential amino acids
eGFR: Estimated glomerular filtration rate
KA: Ketoacids
LPD: Low protein diet (usually 0.6 g prot/kg/d)
MHD: Maintenance hemodialysis
SLPD: Supplemented low protein diet
SVLPD: Supplemented very low protein diet
VLPD: Very low protein diet (usually 0.3–0.4 g prot/kg/d)
